# Regional Differences in the Contributions of TNF Reverse and Forward Signaling to the Establishment of Sympathetic Innervation

**DOI:** 10.1002/dneu.22680

**Published:** 2019-04-30

**Authors:** Clara Erice, O. Yipkin Calhan, Lilian Kisiswa, Sean Wyatt, Alun M. Davies

**Affiliations:** ^1^ School of Biosciences Cardiff University Museum Avenue Cardiff CF10 3AT Wales; ^2^ Present address: Sandra Rotman Centre for Global Health University Health Network: Toronto General Hospital Toronto Ontario Canada; ^3^ Present address: Department of Physiology National University of Singapore Singapore 117597 Singapore

**Keywords:** TNF, TNFR1, sympathetic neuron, sympathetic innervation, development

## Abstract

Members of the TNF and TNF receptor superfamilies acting by both forward and reverse signaling are increasingly recognized as major physiological regulators of axon growth and tissue innervation in development. Studies of the experimentally tractable superior cervical ganglion (SCG) neurons and their targets have shown that only TNF reverse signaling, not forward signaling, is a physiological regulator of sympathetic innervation. Here, we compared SCG neurons and their targets with prevertebral ganglion (PVG) neurons and their targets. Whereas all SCG targets were markedly hypoinnervated in both TNF‐deficient and TNFR1‐deficient mice, PVG targets were not hypoinnervated in these mice and one PVG target, the spleen, was significantly hyperinnervated. These *in vivo* regional differences in innervation density were related to *in vitro* differences in the responses of SCG and PVG neurons to TNF reverse and forward signaling. Though TNF reverse signaling enhanced SCG axon growth, it did not affect PVG axon growth. Whereas activation of TNF forward signaling in PVG axons inhibited growth, TNF forward signaling could not be activated in SCG axons. These latter differences in the response of SCG and PVG axons to TNF forward signaling were related to TNFR1 expression, whereas PVG axons expressed TNFR1, SCG axons did not. These results show that both TNF reverse and forward signaling are physiological regulators of sympathetic innervation in different tissues.

**Abbreviations**
iDISCO: immunolabeling‐enabled three‐dimensional imaging of solvent‐cleared organs
NGF: nerve growth factor
PVG: prevertebral ganglion
SCG: superior cervical ganglion
TH: tyrosine hydroxylase
TNF: tumor necrosis factor
TNFR1: tumor necrosis factor receptor‐1
TNFR2: tumor necrosis factor receptor‐2
TNFRSF: tumor necrosis factor receptor superfamily
TNFSF: tumor necrosis factor superfamily

## Introduction

A variety of extracellular signals regulate the establishment of sympathetic innervation in the developing peripheral nervous system (Glebova and Ginty, 2005; Davies, 2009). The best understood of these is nerve growth factor (NGF), a secreted protein synthesized in tissues innervated by NGF‐responsive neurons. NGF promotes the survival of developing sympathetic neurons, and the level of NGF synthesis in different tissues regulates the number of neurons that innervate these tissues by restricting the extent of cell death among the innervating neurons (Crowley *et al.*, 1994; Davies, 2003). Although NGF plays no role in guiding sympathetic axons to their targets (Davies *et al.*, 1987), it performs the crucial function of promoting the growth and branching of sympathetic axons within most but not all targets of NGF‐dependent sympathetic neurons (Glebova and Ginty, 2004).

A recently discovered target‐derived signal that also promotes sympathetic axon growth and branching during the stage in development when sympathetic axons are ramifying in their targets is tumor necrosis factor receptor 1 (TNFR1). Studies supporting this idea were carried out on the experimentally tractable sympathetic neurons of the superior cervical ganglion (SCG) and their cranial innervation targets. TNFR1 is expressed by SCG targets and acts as a ligand for membrane‐integrated TNF expressed along SCG axons. TNFR1‐activated TNF‐mediated reverse signaling enhances SCG axon growth and branching *in vitro* during the stage when SCG axons are ramifying in their target tissues. Mice lacking either TNF or TNFR1 display greatly reduced sympathetic innervation density *in vivo* in all the SCG target tissues that were studied, but unlike NGF‐deficient mice, these mice showed no deficits in sympathetic neuron number (Kisiswa *et al.*, 2013). While TNF‐activated TNFR1‐mediated forward signaling is able to reduce NGF‐promoted axon growth from dissociated SCG neurons *in vitro* (Gutierrez *et al.*, 2008; Kisiswa *et al.*, 2013), the physiological significance of this *in vitro* inhibitory response of SCG neurons to TNF has remained enigmatic because of the observed hypoinnervation phenotype of SCG targets in TNF‐deficient and TNFR1‐deficient mice.

Here, we have extended the *in vivo* analysis of TNF‐deficient and TNFR1‐deficient mice to additional SCG targets and to prevertebral ganglion (PVG) targets, and have also studied the innervating populations of sympathetic neurons *in vitro*. We find that PVG targets do not exhibit the pronounced hypoinnervation phenotype of SCG targets observed in TNF‐deficient and TNFR1‐deficient mice and show that this *in vivo* difference can be accounted by differences in the response of SCG and PVG neurons to TNF reverse and forward signaling *in vitro*. Our results demonstrate that distinctive signaling mechanisms in different populations of developing sympathetic neurons regulate and refine innervation in different tissues.

## Materials and Methods

### Mice

Breeding and housing of mice (*Mus musculus*) was approved by the Cardiff University Ethical Review Board and was performed within the guidelines of the Home Office Animals (Scientific Procedures) Act, 1986. Mice with null mutations in the *Tnf* gene (Korner *et al.*, 1997) and the *Tnfrsf1a* gene (Pfeffer *et al.*, 1993) in a C57BL/6 background were used. *Tnf^+/−^* mice were crossed to generate litters of* Tnf^+/+^*, *Tnf^+/−^*, and *Tnf^−/−^* pups and *Tnfrsf1a^+/−^* mice were crossed to generate litters of *Tnfrsf1a^+/+^*, *Tnfrsf1a^+/−^*, and *Tnfrsf1a^−/−^* pups. Dissociated cultures of SCG and PVG neurons were established from CD1 mice.

### Quantification of the Sympathetic Innervation

A variety of tissues and organs (pineal gland, trachea, stomach, and spleen) were dissected and processed for visualization of sympathetic fibers by tyrosine hydroxylase (TH) staining in either whole mount tissue preparations or immunolabeling‐enabled three‐dimensional imaging of solvent‐cleared organs (iDISCO) or both. The whole mounts were carried out on tissue from P10 pups as described (Kisiswa *et al.*, 2013) and the iDISCO preparations were carried out on tissue from P6 pups as described (Renier *et al.*, 2014). Batches of tissue from littermates of all three genotypes of each mouse mutant were processed at the same time to ensure they were stained in an identical manner.

For the whole mount preparations, TH‐positive fibers were visualized by HRP‐DAB staining and the specimens were imaged by phase contrast light microscopy (Nikon Diaphot). The same orientation and region was imaged for each organ using the following standard anatomical landmarks: pineal gland, TH‐positive axon bundles entering the gland; trachea, TH‐positive lateral axon bundles entering this structure; and spleen, TH‐positive axon bundles accompanying the splenic arteries. Because it was difficult to maintain the structural integrity of the stomach after removal of milk, the most intensely innervated region closest to the lesser curvature was imaged. To compare the extent of sympathetic nerve branching, a modified line intercept method was used. Using ImageJ, a grid of 24 squares (4 × 6 squares, 158 µm side length per square) was aligned in a standard orientation on images of the trachea, spleen, and stomach. For the smaller pineal gland, the grid consisted of 6 × 6 squares of side length 50 μm per square. Fiber density was estimated using the formula πDI/2, where D is the interline interval in microns (158 or 50) and I is the mean number of fiber bundles intersecting along one side of each grid square. The data are expressed as a percentage of the mean of the wild‐type data. All imaging and quantification was performed blind.

For iDISCO preparations, TH‐positive fibers were fluorescently labeled using anti‐TH antibody (1:200 dilution of antibody AB152, Milliopore, Dundee, UK) and Alexa Fluor 594 secondary antibody (1:300, Abcam). Using a 3D printed compartment, the stomach and spleen were imaged in their entirety using inverted and confocal microscopy (Zeiss Axio Z2 Imager and Zeiss LSM 710) and z‐stacks were constructed. Fiji‐Image J was used for semi‐automated quantification of TH‐immunoreactivity as a measure of sympathetic target organ innervation density. Images were converted to grayscale and the Feature Extraction (FeatureJ Hessian) tool was employed using the smallest eigenvalue of Hessian tensor with the smoothing scale set to 0.5. To ensure consistent analysis across all conditions, multiple images from all mice were initially analyzed to generate a uniform threshold value, which was applied to every image of the same organ analyzed followed by a user‐defined macro to provide quantitative measurement of TH‐immunoreactivity within each target organ. The data are expressed as a percentage of the mean of the wild‐type data. All imaging and quantification was performed blind.

### Dissociated Neuron Culture

Dissected P0 and P5 SCG and prevertebral coeliac and superior mesenteric ganglia of CD1 mice were freed of adherent connective tissue using tungsten needles and were trypsinized and plated at very low density (~200 neurons per dish/well) in poly‐ornithine and laminin‐coated 35‐mm tissue culture dishes (Greiner, Gloucestershire, UK) or 4‐well dishes (Starlab, Milton Keynes, UK) in serum‐free Hams F14 medium (Davies *et al.*, 1993) supplemented with 0.25% Albumax I (Invitrogen, Paisley, UK). Neurons were grown with either 10 ng/ml NGF or 10 ng/ml NGF plus either 30 ng/ml TNFR1‐Fc (R&D Systems) or 50 ng/ml recombinant mouse TNFα (R&D Systems) or 50 ng/ml TNFR2‐Fc (R&D Systems). Analysis of the size and complexity of neurite arbors was carried out in 35 mm dishes 18 h after plating. The neurite arbors were labeled by incubating the neurons with the fluorescent vital dye calcein‐AM (1:1000, Invitrogen, Paisley, UK) at the end of the experiment. Images of neurite arbors were acquired by fluorescence microscopy and analyzed to obtain the Sholl profiles (Gutierrez and Davies, 2007).

### Compartment Cultures

Either SCG or PVG neurons were plated at high density in one compartment (the soma compartment) of a two‐compartment microfluidic device (SND150, Xona microfludics, CA, USA) (Nagendran *et al.*, 2018). At the time of plating, both compartments received NGF plus caspase inhibitor III (Merck, 218745‐1MG). Either TNF or TNFR1‐Fc was added to either the soma or axon compartment or neither compartment received any further treatment. After 18‐h incubation, the soma that projected axons into the axon compartment was labeled by adding the fluorescent vital dye calcein‐AM to the axon compartment. The dye was retrogradely transported and only labeled cell bodies that projected axons into the axon compartment, permitting normalization of axon length to projecting soma number. Very similar high‐density plating was used for all experimental conditions. Axon length was quantified by a modification of a previously described method (Ronn *et al.*, 2000). Briefly, using NIH ImageJ, a grid of vertical lines was constructed with an interline interval of 200 μm. Total intersections between axons and the grid were counted and normalized against the number of labeled somas in the cell body compartment. Average axon length per projecting cell body was calculated using the formula *L* = *DI*/2, where *L* is the estimated length, *D* is the interline interval, and *I* is the average number of intersections per projecting cell body. Measurements were independently carried out in all fields along the microfluidic barrier.

### Immunocytochemistry

The cultures were fixed in 4% paraformaldehyde (PFA) for 20 min and were washed with phosphate‐buffered saline (PBS) before blocking nonspecific binding and permeablizing the cells with 5% bovine serum albumin (BSA) and 5% donkey serum plus 0.1% Triton X‐100 (Sigma, Dorset, UK) in PBS for 1 h at room temperature. Neurons were incubated overnight with primary antibody in 1% blocking solution at 4^o^C. After washing with PBS, the cultures were incubated with the appropriate secondary antibody. The primary antibodies were: anti‐βIII tubulin (1:1500, Abcam, ab41489), anti‐TNF alpha (1:500, Abcam, ab34674), anti‐TNFR1 (1:500, Abcam, ab19139), and anti‐TNFR2 (1:40, R&D, AF‐426‐PB). Secondary antibodies were Alexa Fluor‐conjugated anti‐immunoglobulin from ThermoFisherScientific used at 1:500 (donkey anti‐rabbit IgG Alexa Fluor 488, A21206; goat anti‐chicken Alexa Fluor 594, A11042; and donkey anti‐goat IgG Alexa Fluor 488, A11055). Images were obtained using a Zeiss LSM 710 confocal microscope.

### Reverse Transcription‐qPCR

The levels of *Tnf* and *Tnfrsf1a* mRNAs were quantified by RT‐qPCR relative to a geometric mean of mRNAs for the house keeping enzymes glyceraldehyde phosphate dehydrogenase (GAPDH) and succinate dehydrogenase (SDHA). Total RNA was extracted from whole SCG, celiac, and mesenteric ganglia at P0 with the RNeasy Mini extraction kit (Qiagen, Crawley, UK), and 5 μl was reverse transcribed for 1 h at 45°C using the AffinityScript kit (Agilent, Berkshire, UK) in a 25‐µl reaction according to the manufacturer's instructions. 2 µl of cDNA was amplified in a 20‐µl reaction volume using Brilliant III ultrafast qPCR master mix reagents (Agilent, Berkshire, UK). QPCR products were detected using dual‐labeled (FAM/BHQ1) hybridization probes specific to each of the cDNAs (MWG/Eurofins, Ebersberg, Germany). The PCR primers were: *Tnf* forward, 5ʹ**‐**TAC TTA GAC TTT GCG GAG‐3ʹ and reverse, 5ʹ**‐**AGA GTA AAG GGG TCA GAG**‐**3ʹ; *Tnfrsf1a* forward, 5ʹ‐TTC CCA GAA TTA CCT CAG**‐**3ʹ and reverse, 5ʹ**‐**AAC TGG TTC TCC TTA CAG**‐**3ʹ; *Gapdh* forward, 5ʹ‐GAG AAA CCT GCC AAG TAT G‐3ʹ and reverse, 5ʹ‐GGA GTT GCT GTT GAA GTC‐3ʹ; *Sdha* forward, 5ʹ‐GGA ACA CTC CAA AAA CAG‐3ʹ and reverse, 5ʹ‐CCA CAG CAT CAA ATT CAT‐3ʹ. Dual‐labeled probes were: *Tnf*, FAM‐CAG GTC TAC TTT GGA GTC ATT GCT C‐BHQ1; *Tnfrsf1a*, FAM‐CAC CGT GTC CTT GTC AGC‐BHQ1; *Gapdh*, FAM‐AGA CAA CCT GGT CCT CAG TGT‐BHQ1; *Sdha*, FAM‐CCT GCG GCT TTC ACT TCT CT‐BHQ1. Forward and reverse primers were used at a concentration of 150 nM each and dual‐labeled probes were used at a concentration of 300 nM. PCR was performed using the Mx3000P platform (Agilent, Berkshire, UK) using the following conditions: 45 cycles of 95°C for 12 s and 60°C for 35 s. Standard curves were generated in every 96‐well plate, for each cDNA for every real‐time PCR run, using serial threefold dilutions of reverse transcribed spleen total RNA (Ambion, Paisley, UK). Four separate dissections were performed.

### Statistics

Statistical comparisons for normally distributed data were performed by independent Student's *t*‐test or one‐way ANOVA followed by Dunnet's *post hoc* test and for non parametric data the Mann–Whitney test or one‐way ANOVA Kruskal–Wallis followed by Dunn's *post hoc* test as indicated.

## Results

### Sympathetic Innervation of SCG Targets in Mice with Null Mutations in the *Tnf* and *Tnfrsf1a* Genes

We have previously reported that the sympathetic innervation of three tissues innervated by the SCG, the iris, submandibular salivary gland, and nasal turbinate tissue is markedly reduced in both TNF‐deficient and TNFR1‐deficient mice compared with wild‐type littermates (Kisiswa *et al.*, 2013). To extend this analysis, we examined additional tissues innervated by the SCG in the current study. We used tyrosine hydroxylase (TH) staining in whole mount tissue preparations (Kisiswa *et al.*, 2013) to visualize sympathetic fibers in mice that were heterozygous or homozygous for null mutations in the *Tnf* gene (Korner *et al.*, 1997), null mutations in the *Tnfrsf1a* gene (Pfeffer *et al.*, 1993), and wild‐type littermates. We carried out this analysis at P10, which is after the period of development during which TNFR1‐activated TNF reverse signaling enhances SCG axon growth *in vitro* and is at a stage *in vivo* when sympathetic innervation has become established (Kisiswa *et al.*, 2013). All analysis was done blind.

**Figure 1:**
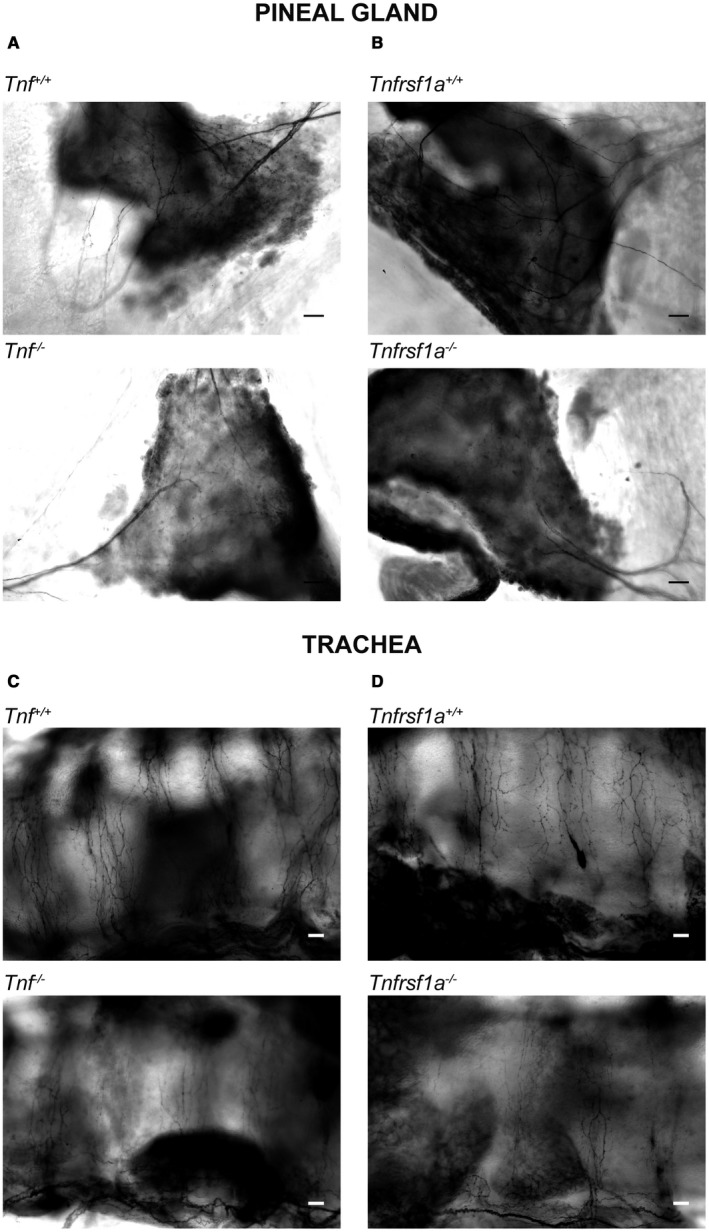
Images of reduced sympathetic innervation in the pineal gland and trachea of mice possessing null mutations in the *Tnf* and *Tnfrsf1a* genes. Representative images of P10 whole mount preparations of the pineal gland (A, B) and trachea (C, D) of P10 *Tnf^−/−^*and *Tnf^+/+^* littermates (A, C) and P10 *Tnfrsf1a^−/−^*and *Tnfrsf1a^+/+^* littermates (B, D) stained with anti‐TH. Scale bars, 100 μm.

**Figure 2:**
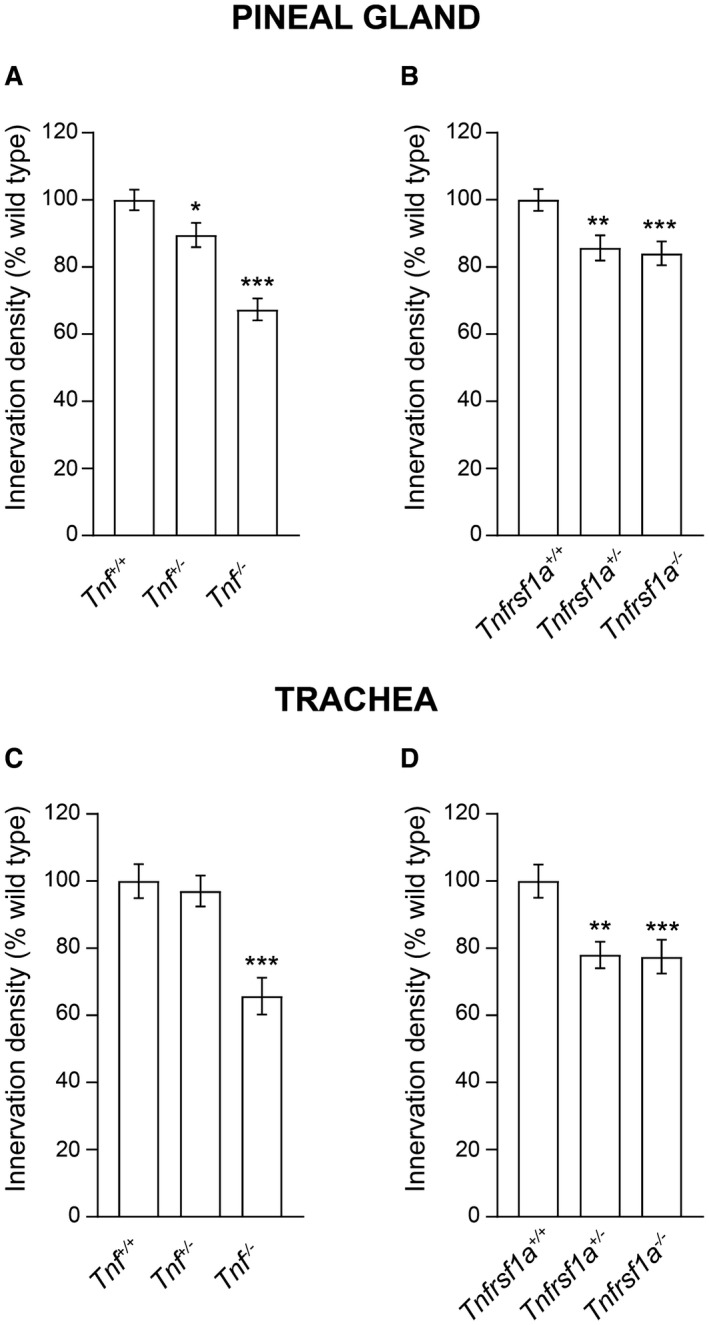
Quantification of reduced sympathetic innervation in the pineal gland and trachea of mice possessing null mutations in the *Tnf* and *Tnfrsf1a* genes. Relative length per unit area of sympathetic fibers in the pineal gland (A, B) and trachea (C, D) of P10 whole mount preparations of wild‐type mice and littermates that are heterozygous or homozygous for null mutations of the *Tnf* gene (A, C) and *Tnfrsf1a* gene (B, D). Data are expressed as a percentage of wild type. Mean ± s.e.m. of data from the pineal gland of six *Tnf^+/+^*_, _seven* Tnf^+/−^*, eight *Tnf^−/−^*, six *Tnfrsf1a^+/+^*_, _six *Tnfrsf1a^+/−^*, seven *Tnfrsf1a^−/−^* animals and from the trachea of five *Tnf^+/+^*_, _six* Tnf^+/−^*, six *Tnf^−/−^*, five *Tnfrsf1a^+/+^*_, _seven *Tnfrsf1a^+/−^*, six *Tnfrsf1a^−/−^* animals (*P < 0.05, **P < 0.01 and ***P < 0.001 compared to wild type, one‐way ANOVA Kruskal–Wallis, Dunn's *post hoc* test).

The additional SCG targets examined were the pineal gland and the trachea. The latter tissue is of particular interest because it is the only tissue in which NGF plays no role in promoting sympathetic fiber ramification during development (Glebova and Ginty, 2004). TH‐stained whole mount preparations revealed clear reductions in the ramification of sympathetic fibers within both tissues in TNF‐deficient and TNFR1‐deficient mice compared with wild‐type littermates. Representative images of both tissues in *Tnf^−/−^* and *Tnf^+/+^* littermates and in *Tnfrsf1a^−/−^* and *Tnfrsf1a^+/+^* littermates are shown in Fig. 1. Using a stereological method to estimate the extent of the growth and branching of sympathetic fibers within these tissues, we found highly significant decreases in *Tnf^−/−^* mice compared with *Tnf^+/+^* littermates and *Tnfrsf1a^−/−^* mice compared with *Tnfrsf1a^+/+^* littermates (Fig. 2). There were also significant reductions in *Tnf^+/−^* and *Tnfrsf1a^+/−^* mice in the pineal gland (Fig. 2A,B) and a significant reduction in the trachea of *Tnfrsf1a^+/−^* mice (Fig. 2D), suggesting gene dosage effects.

### Sympathetic Innervation of PVG Targets in Mice with Null Mutations in the *Tnf* and *Tnfrsf1a* Genes

We examined two organs that receive their sympathetic innervation predominantly from PVG neurons, the stomach, and spleen. The stomach receives its sympathetic innervation predominantly from the coeliac and superior mesenteric ganglia and has smaller contributions from the splanchnic ganglia and the lower thoracic paravertebral sympathetic chain. The spleen receives its sympathetic innervation predominantly from the splanchnic ganglia and has smaller contributions from the inferior mesenteric and lower thoracic paravertebral sympathetic chain (Chevendra and Weaver, 1991; Trudrung *et al.*, 1994; Quinson *et al.*, 2001).

In addition to using TH staining in whole mount tissue preparations to visualize sympathetic fibers at P10 (Kisiswa *et al.*, 2013), we also used TH staining in iDISCO (Renier *et al.*, 2014). For technical reasons, the latter analysis was carried out at P6, which is still after sympathetic innervation has become established (Glebova and Ginty, 2004). As with analysis of SCG targets, all quantification was done blind.

**Figure 3:**
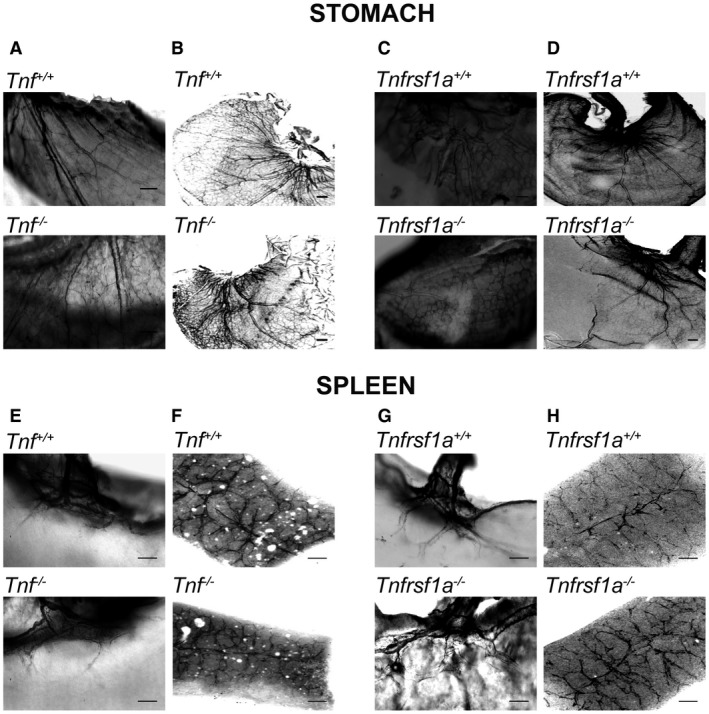
Images of sympathetic innervation of the stomach and spleen of mice possessing null mutations in the *Tnf* and *Tnfrsf1a* genes. Representative images of P10 whole‐mount (A, C) and P6 iDISCO (B, D) preparations of the stomach and of P10 whole‐mount (E, G) and P6 iDISCO (F, H) preparations of the spleen of *Tnf^−/−^* and *Tnf^+/+^* littermates (A, B, E, F) and *Tnfrsf1a^−/−^*and *Tnfrsf1a^+/+^* littermates (C, D, G, H) stained with anti‐TH. Scale bars, 200 μm.

**Figure 4:**
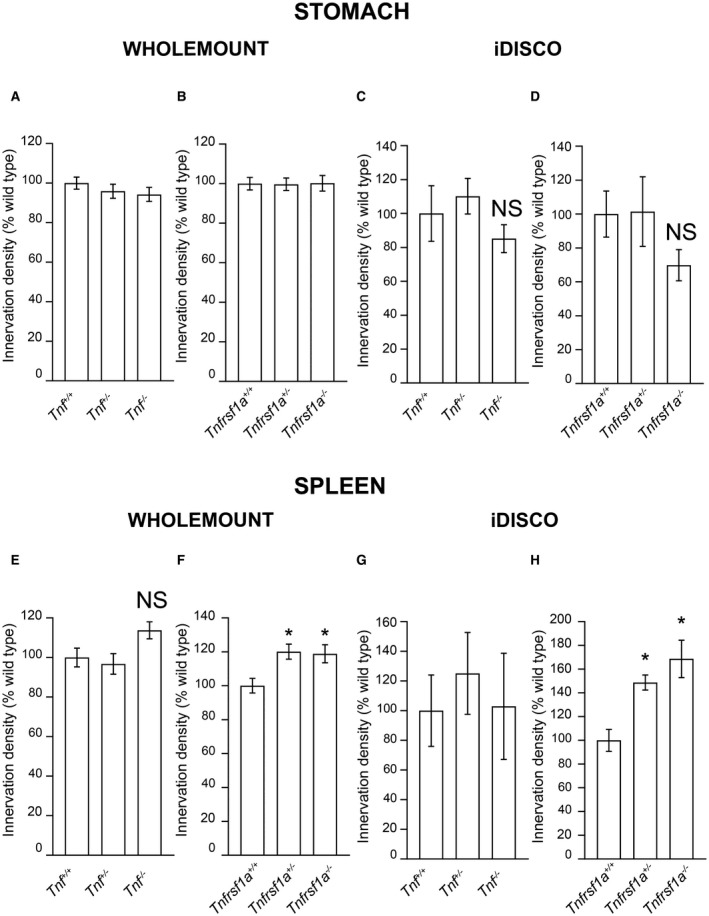
Quantification of sympathetic innervation of the stomach and spleen of mice possessing null mutations in the *Tnf* and *Tnfrsf1a* genes. Relative length per unit area of TH‐positive fibers in stomach whole mounts (A, B) and relative TH immunofluorescence intensity in stomach iDISCO preparations (C, D) of wild type mice and littermates that are heterozygous or homozygous for null mutations of the *Tnf* gene (A, C) and *Tnfrsf1a* gene (B, D). Relative length per unit area of TH‐positive fibers in spleen whole mounts (E, F) and relative TH immunofluorescence intensity in spleen iDISCO preparations (G, H) of wild‐type mice and littermates that are heterozygous or homozygous for null mutations of the *Tnf* gene (E, G) and *Tnfrsf1a* gene (F, H). Data are expressed as a percentage of wild type. Mean ± s.e.m. of data from the stomach of six *Tnf^+/+^*_, _siz* Tnf^+/−^*, six *Tnf^−/−^*, eight *Tnfrsf1a^+/+^*_, _eight *Tnfrsf1a^+/−^*, five *Tnfrsf1a^−/−^* animals and from the spleen of six *Tnf^+/+^*_, _eight* Tnf^+/−^*, seven *Tnf^−/−^*, eight *Tnfrsf1a^+/+^*_, _eight *Tnfrsf1a^+/−^*, six *Tnfrsf1a^−/−^* animals in whole mount preparations. Mean ± s.e.m. of data from the stomach of eight *Tnf^+/+^*_, _eight* Tnf^+/−^*, eight *Tnf^−/−^*, nine *Tnfrsf1a^+/+^*_, _seven *Tnfrsf1a^+/−^*, eight *Tnfrsf1a^−/−^* animals and from the spleen of eight *Tnf^+/+^*_, _eight* Tnf^+/−^*, nine *Tnf^−/−^*, nine *Tnfrsf1a^+/+^*_, _nine *Tnfrsf1a^+/−^*, six *Tnfrsf1a^−/−^* animals in iDISCO preparations. *P < 0.05 and NS = not significant, compared to wild type, one‐way ANOVA for iDISCO stomach and Kruskal–Wallis, Dunn's *post hoc* test all the rest.

There was no obvious difference in the anatomical disposition of sympathetic fibers in the stomach of *Tnf^−/−^* and *Tnfrsf1a^−/−^* mice compared with *Tnf^+/+^* and *Tnfrsf1a^+/+^* littermates. Representative whole mount and iDISCO images of the stomach of *Tnf^−/−^* and *Tnf^+/+^* littermates and *Tnfrsf1a^−/−^* and *Tnfrsf1a^+/+^* littermates are shown in Fig. 3A–D. Quantification of the relative length per unit area of TH‐positive fibers in whole mount preparations (Fig. 4A,B) and TH‐intensity in iDISCO preparations (Fig. 4C,D) confirmed that there was no significant reduction in sympathetic fiber branching within the stomach between *Tnf^−/−^* and *Tnf^+/+^* littermates (Fig. 4A,C) and between *Tnfrsf1a^−/−^* and *Tnfrsf1a^+/+^* littermates (Fig. 4B,D).

There was also no obvious difference in the anatomical disposition of sympathetic fibers in the spleen of *Tnf^−/−^* and *Tnfrsf1a^−/−^* mice compared with *Tnf^+/+^* and *Tnfrsf1a^+/+^* littermates. Representative whole mount and iDISCO images of the spleen of *Tnf^−/−^* and *Tnf^+/+^* littermates and *Tnfrsf1a^−/−^* and *Tnfrsf1a^+/+^* littermates are shown in Fig. 3E–H. Quantification of the relative length per unit area of TH‐positive fibers in whole mount preparations (Fig. 4E,F) and TH‐intensity in iDISCO preparations (Fig. 4G,H) confirmed that there was no significant reduction in sympathetic fiber branching within the spleen between *Tnf^−/−^* and *Tnf^+/+^* littermates (Fig. 4E,G) and between *Tnfrsf1a^−/−^* and *Tnfrsf1a^+/+^* littermates (Fig. 4F,H). There were, however, small statistically significant increases in the innervation of the spleen in mice that were heterozygous and homozygous for a null mutation in the *Tnfrsf1a* gene quantified in both whole mount and iDISCO preparations (Fig. 4F,H).

### Differences in the Response of SCG and PVG Neurons to TNFR1‐Activated TNF Reverse Signaling

To determine if the different effects of *Tnf* and *Tnfrsf1a* deletion on the innervation of SCG and PVG targets were due to differences in the response of SCG and PVG neurons to TNFR1‐activated TNF reverse signaling we cultured postnatal SCG and PVG neurons at low density in dissociated cultures. NGF was included in these cultures to sustain neuronal survival and the neurons were treated with and without a divalent TNFR1‐Fc chimera (in which the extracellular domains of two TNFR1 molecules are linked to the Fc part of a human IgG1 antibody) to activate TNF‐mediated reverse signaling (Kisiswa *et al.*, 2013). We studied the PVG neurons of the coeliac and superior mesenteric ganglia, because these ganglia can be most easily dissected from neonates.

**Figure 5:**
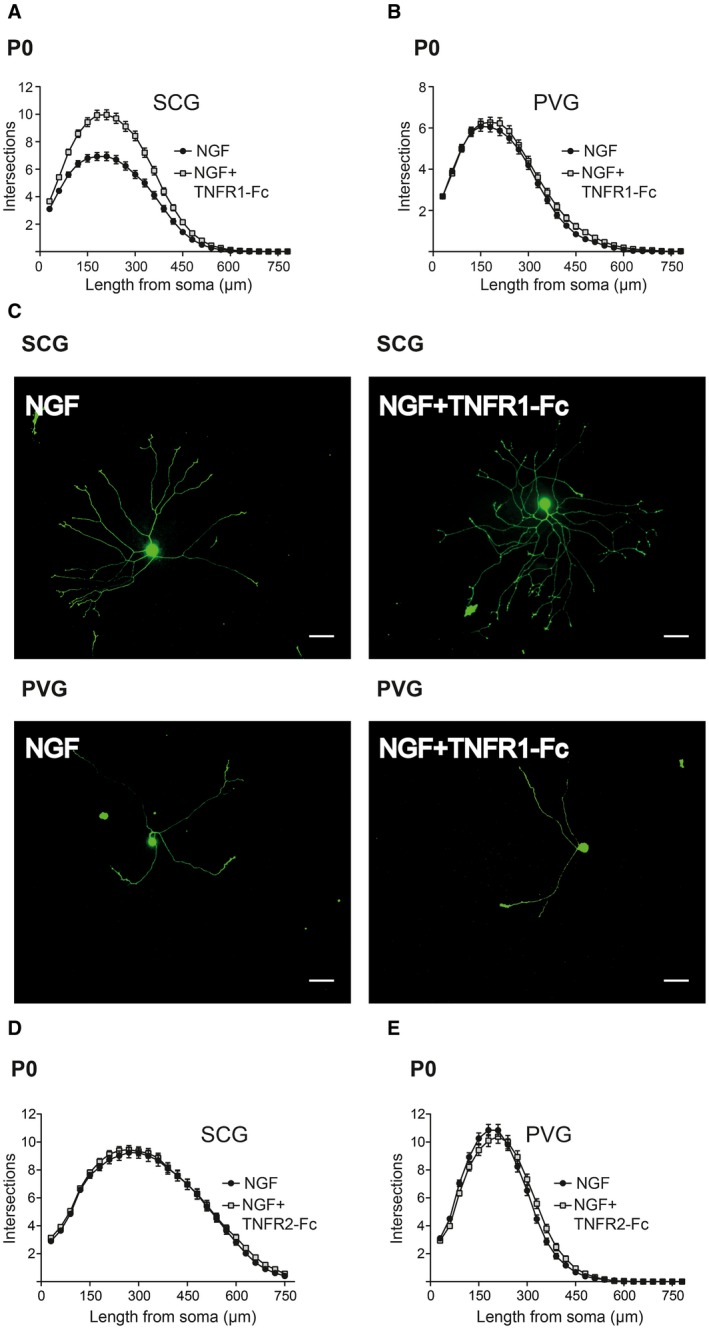
Differences in the response of cultured SCG and PVG neurons to TNFR1‐activated TNF‐mediated reverse signaling. Sholl plots of P0 SCG (A) and PVG (B) neurons cultured for 18 h with either 10 ng/ml NGF or NGF plus 30 ng/ml TNFR1‐Fc. Mean ± s.e.m. of data of >150 neurons per condition combined from three separate experiments of each type. (C) Images of representative P0 SCG neurons and PVG neurons grown for 18 h with NGF with or without TNFR1‐Fc as indicated. Scale bar, 50 μm. Sholl plots of P0 SCG (D) and PVG (E) neurons cultured for 18 h with either 10 ng/ml NGF or NGF plus 50 ng/ml TNFR2‐Fc. Mean ± s.e.m. of data of >150 neurons per condition combined from three separate experiments of each type. [Colour figure can be viewed at wileyonlinelibrary.com]

The axon growth‐promoting action of NGF on P0 SCG neurons was enhanced by TNFR1‐Fc, as shown by Sholl analysis in P0 cultures (Fig. 5A). In contrast, TNFR1‐Fc failed to enhance axon growth and branching from P0 PVG neurons (Fig. 5B). Sholl analysis carried out at P5 revealed a similar trend (not shown). Representative images of SCG and PVG neurons grown with and without TNFR1‐Fc are shown (Fig. 5C). These findings suggest that TNFR1‐activated TNF‐mediated reverse signaling is able to enhance axon growth from postnatal SCG neurons but not from postnatal PVG neurons.

### TNFR2‐Fc Does Not Influence Axon Growth from Either SCG or PVG Neurons

Because TNFR2 has been shown to elicit TNF‐mediated reverse signaling in the immune system (Sun and Fink, 2007), we investigated whether TNFR2‐activated TNF reverse signaling might differentially influence axon growth from SCG and PVG neurons. TNFR2‐Fc did not significantly affect axon growth from either SCG or PVG neurons grown with NGF (Fig. 5D,E).

### TNF Suppresses NGF‐Promoted Growth from Both SCG and PVG Neurons

**Figure 6:**
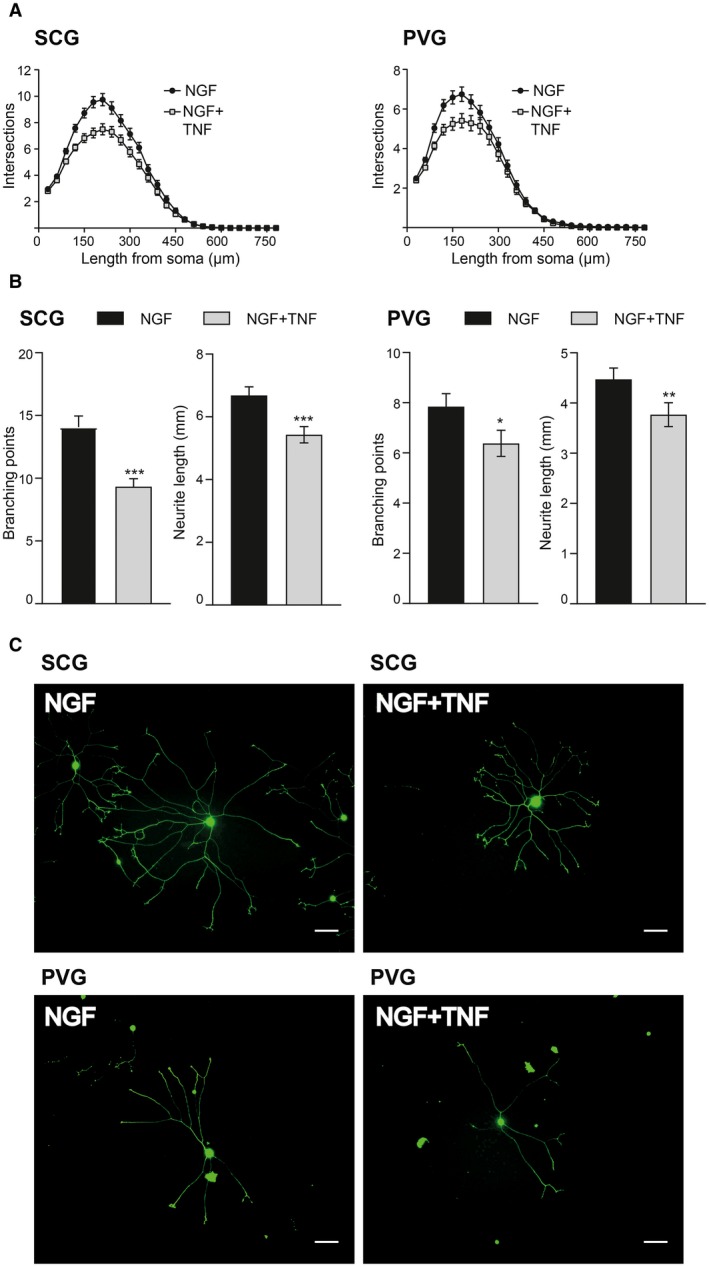
TNF suppresses NGF‐promoted axon growth from both SCG and PVG neurons in dissociated cultures. Sholl plots (A) and quantification of number of branch points and the total axon length (B) of the arbors of P0 SCG and PVG neurons cultured for 18 h with either 10 ng/ml NGF or NGF plus 50 ng/ml TNF. Mean ± s.e.m. of data of >150 neurons per condition combined from three separate experiments of each type (*** indicates *P* < 0.001, ** indicates *P* < 0.01, * indicates *P* < 0.05, Mann–Whitney). (C) Images of representative SCG neurons and PVG neurons grown for 18 h with NGF with or without TNF as indicated. Scale bar, 50 μm. [Colour figure can be viewed at wileyonlinelibrary.com]

Soluble TNF is known to suppress NGF‐promoted axon growth from cultured SCG neurons (Gutierrez and Davies, 2007). Because suppression of axon growth by TNF is not observed in TNFR1‐deficient neurons (Kisiswa *et al.*, 2013), it is likely that soluble TNF acts by a TNFR1‐mediated forward signaling mechanism. To ascertain whether TNF has a comparable effect on NGF‐promoted axon growth from PVG neurons, we compared the effect of TNF on NGF‐promoted axon growth from SCG and PVG neurons in low‐density dissociated cultures at P0. TNF suppressed NGF‐promoted axon growth from both SCG and PVG neurons. This was evident in Sholl plots of both kinds of neurons (Fig. 6A). Quantification of the total length and number of branch points in the axon arbors of SCG and PVG neurons revealed statistically significant decreases in length and branching in the presence of TNF in both kinds of neurons (Fig. 6B). Sholl analysis carried out at P5 revealed a similar trend (not shown). The appearances of typical SCG and PVG neurons grown with and without TNF are shown (Fig. 6C). These findings suggest that TNF suppresses NGF‐promoted axon growth from SCG neurons and PVG neurons grown in dissociated culture in which both the soma and axons of these neurons are exposed to TNF.

### TNF Suppresses NGF‐Promoted Growth When Applied to Different Regions of SCG and PVG Neurons in Compartment Culture

**Figure 7:**
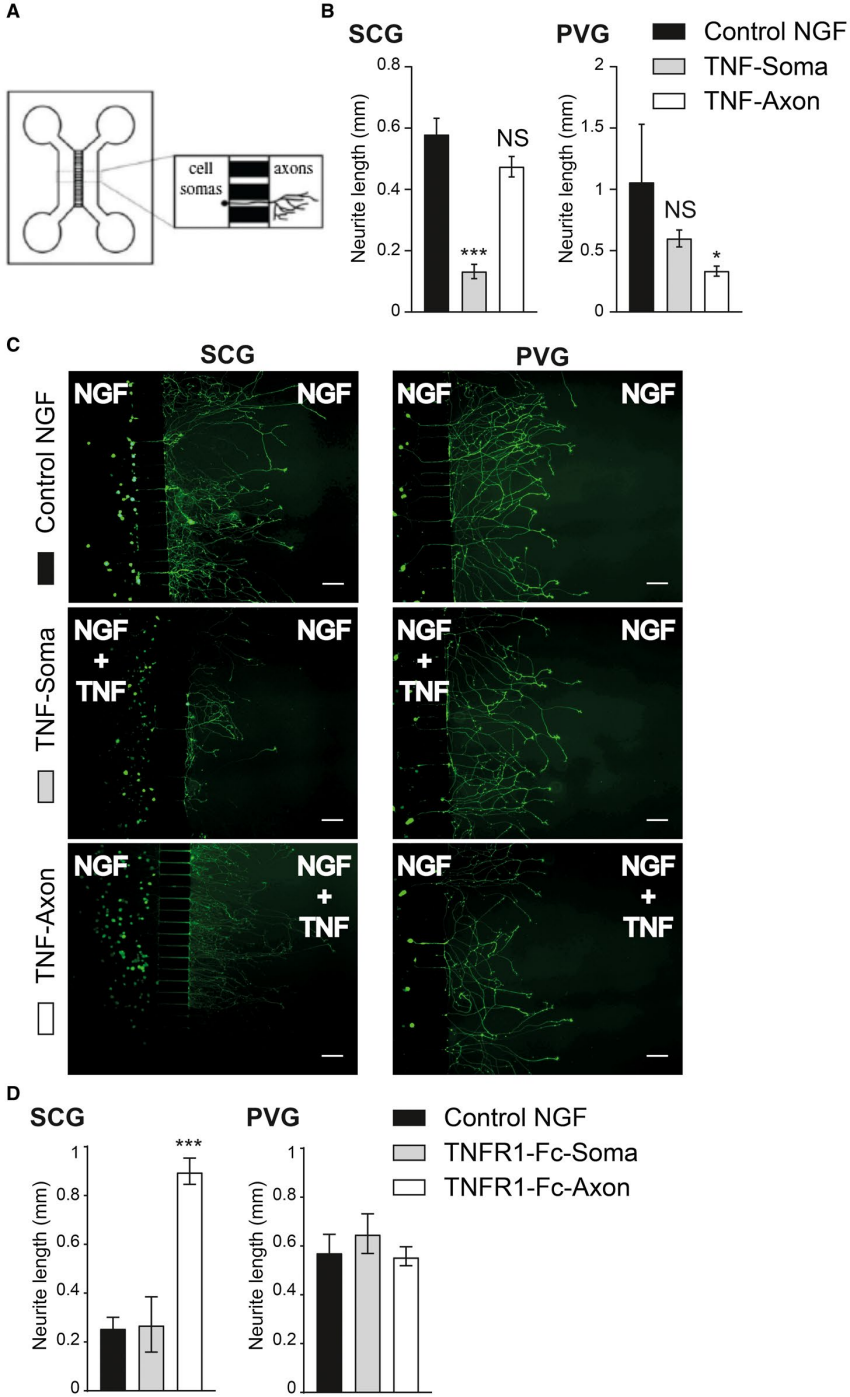
TNF suppresses NGF‐promoted growth when applied to PVG axons, but not SCG axons. (A) Schematic illustration of the two‐chamber microfluidic device. (B) Bar charts of mean axon length of either P0 SCG or P0 PVG neurons projecting axons into the axon compartment quantified after 18‐h incubation. Both compartments contained 10 ng/ml NGF plus caspase inhibitor and the compartments either received no additional treatment (control) or 50 ng/ml TNF was added to either the soma or axon compartment. The data represent the mean ± s.e.m of 3 independent experiments (*** indicates *P* < 0.001, * indicates *P* < 0.05, and NS indicates not significant, statistical comparison with control, one‐way ANOVA Kruskal–Wallis, Dunn's *post hoc*) (C) Representative images of calcein‐AM labeled neurons cultured under the experimental conditions labeled. Scale bar, 100 μm. (D) Bar charts of mean axon length of either P0 SCG or P0 PVG neurons projecting axons into the axon compartment quantified after 18 h incubation. Both compartments contained 10 ng/ml NGF plus caspase inhibitor and the compartments either received no additional treatment (control) or 50 ng/ml TNFR1‐Fc was added to either the soma or axon compartment. The data represent the mean ± s.e.m of 1 experiment for SCG neurons and three independent experiments for PVG neurons (*** indicates *P* < 0.001, * indicates *P* < 0.05, and NS indicates not significant, statistical comparison with control, one‐way ANOVA Kruskal–Wallis, Dunn's *post hoc*). [Colour figure can be viewed at wileyonlinelibrary.com]

Although TNF suppressed NGF‐promoted axon growth from both SCG and PVG neurons when the axons and cell soma of these neurons were exposed to TNF *in vitro*, because target‐derived TNF acts only on axons *in vivo*, we investigated whether SCG and PVG neurons differ in their response to TNF when treatment is restricted to axons. To do this, we grew the SCG and PVG neurons in compartment cultures in which the cell soma and axons are separated by a barrier (Fig. 7A). In these experiments, we seeded either P0 SCG or P0 PVG neurons into one compartment (the soma compartment) of a two‐compartment device that contained NGF in both compartments to sustain neuronal survival and encourage axon growth from the soma compartment into the axon compartment. TNF was added either to the soma compartment or to the axon compartment or to neither compartment. After 18‐h incubation, we labeled the axons in the axon compartment with the fluorescent vital dye calcein‐AM, which also retrogradely labeled cell bodies of neurons that projected axons into the axon compartment, allowing for normalization of axon length against cell body number. A stereological method was used to quantify the extent of axon growth in the axon compartment relative to the number of neurons projecting axons into this compartment.

These compartment cultures revealed a clear difference in the response of SCG and PVG neurons to TNF. Whereas exposure of the cell soma of SCG neurons to TNF significantly reduced axon growth in the axon compartment, exposure of SCG axons to TNF did not significantly affect axon growth (Fig. 7B,C). In marked contrast, whereas exposure of the cell soma of PVG neurons to TNF did not significantly affect axon growth in the axon compartment, exposure of PVG axons to TNF significantly reduced axon growth (Fig. 7B,C). These results reveal that TNF significantly suppresses axon growth when applied to different parts of SCG and PVG neurons.

Although TNFR1‐Fc did not affect PVG axon growth *in vitro*, we investigated the possibility of it exerting effects when applied to different regions of PVG neurons. As previously reported (Kisiswa *et al*, 2013), TNFR1‐Fc increased SCG axon length when applied to axons and not somas (Fig. 7D). On the other hand, in three independent experiments. TNFR1‐Fc did not significantly affect axon growth from PVG neurons when applied to either the soma or axon compartments (Fig. 7D).

### Expression of TNF and TNFR1 in SCG and PVG Neurons

**Figure 8:**
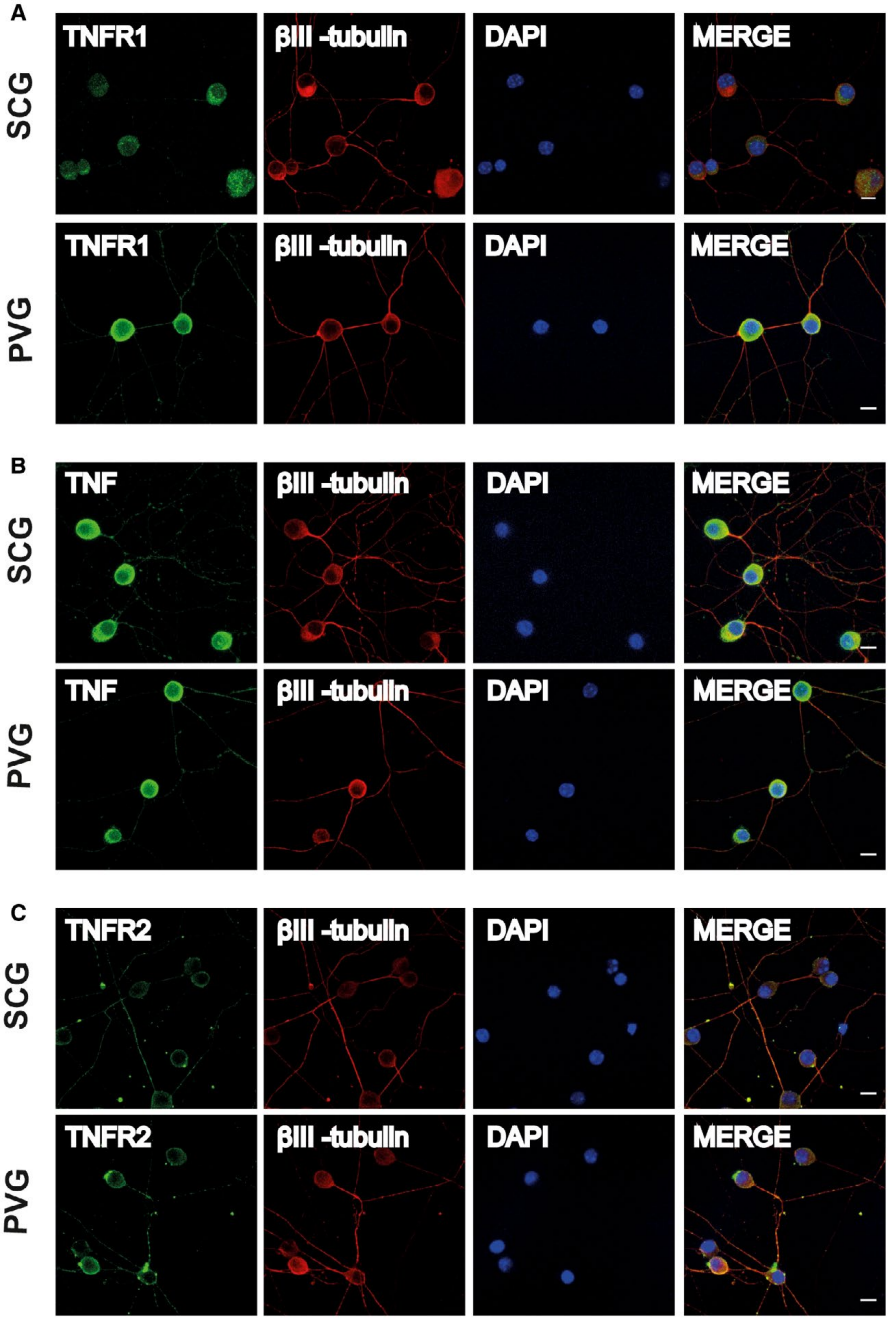
Immunolocalization of TNF, TNFR1, and TNFR2 on SCG and PVG neurons. Representative photomicrographs of P0 SCG neurons and PVG neurons double labeled with either anti‐TNFR1 and anti‐βIII‐tubulin antibodies (A) or anti‐TNF and anti‐βIII‐tubulin antibodies (B) or anti‐TNFR2 and anti‐βIII‐tubulin antibodies (C) after 24‐h culture in NGF‐supplemented medium. The preparations were also counterstained with the nuclear marker, DAPI. Scale bars, 10 μm. [Colour figure can be viewed at wileyonlinelibrary.com]

A potential explanation for the differences in the response of SCG and PVG neurons to TNF in compartment cultures might be related to differences in the expression and/or the distribution of TNFR1 and TNF on these neurons. It has been reported that the TNFR1 expression is restricted to the cell body of SCG neurons and is not detectable on the axons of these neurons, accounting for the lack of response of SCG neurons to TNF applied to their axons (Kisiswa *et al.*, 2013). To ascertain whether TNFR1 expression on PVG neurons has a different spatial distribution that could account for the response of these neurons to TNF applied to their axons, we used immunocytochemistry to study the expression of TNFR1 on dissociated cultured neurons. To label both the cell soma and axons of the neurons, the cultures were double labeled with βIII‐tubulin antibodies. As previously reported, TNFR1 immunoreactivity was restricted to the cell soma of SCG neurons and was not evident on their axons (Fig. 8A). In contrast, in PVG cultures set up and processed in parallel, TNFR1 immunoreactivity was observed along the axons as well as the cell soma (Fig. 8A). This difference in the distribution of TNFR1 between SCG and PVG neurons is the likely explanation of the ability of the PVG neurons, but not SCG neurons, to respond to TNF applied to their axons. This in turn implies that PVG neurons, but not SCG neurons, are capable of responding to target‐derived TNF *in vivo*.

Like TNFR1 immunoreactivity, TNF immunoreactivity was evident on both SCG and PVG neurons. However, in contrast to TNFR1 immunoreactivity, TNF immunoreactivity was observed on the cell soma and the axons of both SCG and PVG neurons (Fig. 8B). Thus, differences in the expression of TNF are unlikely to account for the lack of a TNF‐mediated axon growth response of PVG neurons to TNFR1. To demonstrate the specificity of the antibodies used in the above studies, we carried out immunocytochemistry on cultures established from knockout animals. TNF immunoreactivity was eliminated in cultures established from *Tnf^−/−^* mice and TNFR1 immunoreactivity was eliminated in cultures established from *Tnfrsf1a^−/−^*mice (not shown).

We also studied and compared TNFR2 immunoreactivity in cultured SCG and PVG neurons using a specific anti‐TNFR2 antibody. As previously reported, TNFR2 immunoreactivity was low compared with TNF and TNFR1 immunoreactivity in SCG neurons (Kisiswa *et al.*, 2013). Using identical staining protocols and the same fluorescence microscope settings, TNFR2 immunoreactivity was markedly lower at cell soma compared with TNF and TNFR1 immunoreactivity and TNFR2 immunoreactivity was only evident along the axons of a subset of SCG neurons (Fig. 8C). A similar low level of TNFR2 immunoreactivity was evident in PVG neuron soma and TNFR2 immunoreactivity was evident along the axons of a similar subset of PVG neurons (Fig. 8C). The similar low level and distribution of TNFR2 immunoreactivity in SCG and PVG neurons suggests that differences in the expression of TNFR2 is unlikely to contribute to the differences observed between SCG and PVG neurons *in vivo* and *in vitro*.

### Expression of TNF and TNFR1 Transcripts in the SCG, PVG, and Their Targets

**Figure 9:**
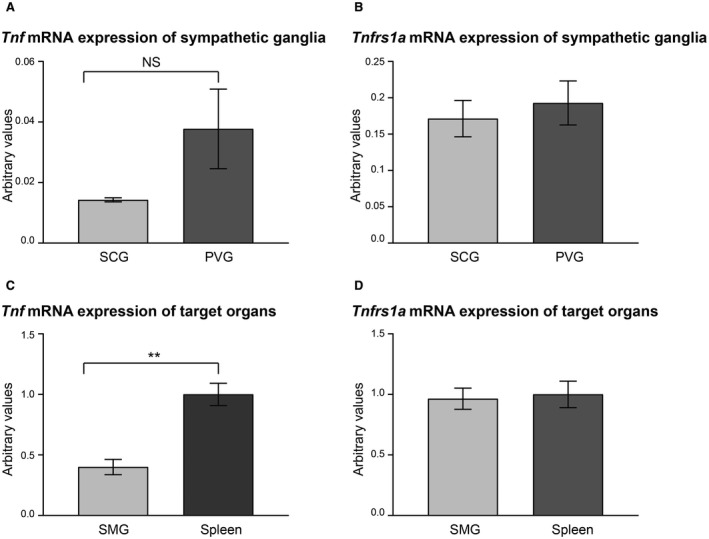
Relative expression of *Tnf* and *Tnfrsf1a* mRNAs in SCG and PVG and their target organs. Bar charts of the levels of *Tnf* mRNA (A) and *Tnfrsf1a* mRNA (B) in P0 SCG and P0 PVG. Bar charts of the levels of *Tnf* mRNA (C) and *Tnfrsf1a* mRNA (D) in P0 submandibular gland (SMG) and P0 spleen. Mean ± s.e.m of data from four separate samples (arbitrary values, ** indicates *P* < 0.01, *t*‐test).

To assess the expression of transcripts encoding TNF and TNFR1 in the SCG and PVG, we used reverse transcription qPCR to quantify the levels of these transcripts relative to the levels of reference mRNAs encoding the house keeping enzymes glyceraldehyde phosphate dehydrogenase and succinate dehydrogenase. Both kinds of ganglia expressed transcripts encoding these proteins (Fig. 9A,B).

To determine whether transcripts encoding TNF and TNFR1 are present in SCG and PVG targets and to compare the relative levels of these transcripts in targets and ganglia, we used qPCR to quantify the levels of these transcripts relative to reference mRNAs in two tissues: the submandibular gland (SMG) and the spleen. *Tnf* and *Tnfrsf1a* mRNAs were present in both tissues, but at much higher levels than in the innervating ganglia. The level of *Tnf* mRNA was about 20‐fold higher in tissue than in the ganglia (Fig. 9C) and the level of *Tnfrsf1a* mRNA was about fivefold higher in tissue then in the ganglia (Fig. 9D). The level of *Tnf* mRNA was significantly higher in the spleen than in the SMG (Fig. 9C) and the levels of *Tnfrsf1a* mRNA were not significantly different in these tissues. These findings imply that TNF and TNFR1 are synthesized in both tissues where they are potentially able to act on the innervating axons.

## Discussion

We have used *in vivo* and *in vitro* approaches to assess the contribution of TNF and TNFR1 to the establishment of sympathetic innervation of different tissues in developing mice and to clarify the underlying signaling mechanisms. Our *in vivo* studies of the phenotypes of TNF‐deficient and TNFR1‐deficient mice have revealed significant decreases in the sympathetic innervation density of SCG targets compared with wild‐type mice but either no change or hyperinnervation of PVG targets. Our *in vitro* studies suggest that these phenotypic differences in innervation are related in part to differences in the specific growth responses of SCG and PVG neurons to TNF and TNFR1 applied to their axons. This implies that anatomically distinct populations of sympathetic neurons differ in their capacity to respond to target‐activated TNF forward and reverse signaling.

Quantification of sympathetic innervation in the pineal gland and trachea of *Tnf^−/−^* and *Tnfrsf1a^−/−^* postnatal mice revealed highly significant 20%–30% reductions compared with wild‐type littermates. Highly significant reductions in sympathetic innervation of similar magnitudes (20%–50% compared with wild‐type littermates) were also reported in three other SCG targets, the submandibular salivary gland, nasal turbinates, and iris, in *Tnf^−/−^* and *Tnfrsf1a^−/−^* postnatal mice (Kisiswa *et al.*, 2013). Significant intermediate reductions in sympathetic innervation were observed in most of these tissues and in the pineal gland and trachea of heterozygous mice, which is consistent with gene dose effects in all tissues. Taken together, our current findings and previously reported findings suggest that TNF and TNFR1 play an important role in establishing the sympathetic innervation of all SCG targets examined. These *in vivo* studies together with the previously reported expression of TNF, but not TNFR1, along SCG axons and the expression of TNFR1 by SCG target field cells (Kisiswa *et al.*, 2013) suggest that TNFR1 expressed in target tissue activates TNF reverse signaling in axons in the target tissue. TNF reverse signaling in turn promotes axon growth by triggering Ca^2+^ influx via T‐type Ca^2+^ channels, which leads to activation of PKC and ERK (Kisiswa *et al.*, 2013, 2017).

Our current demonstration of the importance of TNF and TNFR1 in the establishment of tracheal sympathetic innervation is especially intriguing because the trachea is the only structure in which sympathetic fibers grow and branch independently of NGF. Mice lacking NGF and BAX, a key protein required for apoptosis, retain sympathetic neurons but have reduced or absent sympathetic innervation in all tissues except the trachea (Glebova and Ginty, 2004). This raised the question of the factor(s) that promote the growth and ramification of sympathetic fibers in this tissue. The substantial significant reductions in sympathetic innervation in the trachea of *Tnf^−/−^* and *Tnfrsf1a^−/−^* mice suggest that TNF and TNFR1, probably by a reverse signaling mechanism, make a significant contribution to the establishment of the sympathetic innervation of this tissue. Tracheal sympathetic innervation is not, however, eliminated in *Tnf^−/−^* and *Tnfrsf1a^−/−^* mice, suggesting that additional factors are involved. The demonstration that tracheal sympathetic innervation is significantly reduced to a small extent in mice lacking GDF5 suggests that this factor also makes a contribution to establishing the sympathetic innervation of this tissue (O'Keeffe *et al.*, 2016).

In marked contrast to all SCG targets examined, there was either no significant decrease in the sympathetic innervation of the spleen and stomach of *Tnf^−/−^* and *Tnfrsf1a^−/−^* mice, tissues that are predominantly innervated by PVG neurons, or small statistically significant increases in the innervation of the spleen in *Tnfrsf1a^−/−^* mice quantified in both whole mount and iDISCO preparations. An increase in sympathetic innervation was also observed in *Tnf^−/−^* mice in whole mount preparations, although this did not reach statistical significance. This implies that TNFR1 at least plays a role in suppressing the ramification of sympathetic axons in the spleen. We are confident of these results because large numbers of mutant and wild‐type mice were examined blind. Moreover, we visualized sympathetic fibers using two methods, whole mount and iDISCO, and studied innervation at two ages, P6 and P10. While there is some difference in the mean increase in innervation of the spleen in *Tnfrsf1a^−/−^* mice compared with *Tnfrsf1a^+/+^* littermates in iDISCO and whole mount preparations, the statistical significance of the increase is similar in both cases. Although it is possible that this difference could be related to the different ages at which analysis was undertaken, sympathetic fibers first reach their targets during early fetal development and have ramified extensively in their target tissues by P6 and P10.

The marked difference between the SCG and PVG targets observed in *Tnf^−/−^* and *Tnfrsf1a^−/−^* mice appears to be related to intrinsic differences in the response of SCG and PVG neurons to TNFR1 and TNF. First, while TNFR1‐Fc significantly enhanced NGF‐promoted axon growth from cultured postnatal SCG neurons, TNFR1‐Fc failed to enhance NGF‐promoted axon growth from PVG neurons of the coeliac and superior mesenteric ganglia. These differences in the response of SCG and PVG neurons to TNFR1‐Fc are not simply due to differences in expression of the reverse signaling receptor. TNF is expressed along the axons of both SCG and PVG axons. Moreover, it is likely that PVG axons are exposed to TNFR1 *in vivo*, as this is expressed in PVG targets. It will be interesting in future work to ascertain which step or steps in the signaling cascade leading from TNF activation to axon growth is blocked in PVG neurons. Second, while TNF suppresses NGF‐promoted axon growth from both SCG and PVG neurons grown in dissociated culture in which both axons and cell soma are exposed to TNF, TNF only suppresses axon growth from PVG neurons, not SCG neurons, when axons are exposed to TNF in compartment cultures. This difference in the response of PVG and SCG neurons in compartment cultures to the growth inhibitory actions of TNF appears to be related to the distribution of TNFR1, the forward signaling receptor that mediates this response, on the neurons. Whereas TNFR1 expression is restricted to the cell soma of SCG neurons, TNFR1 is expressed both at the cell soma and along the axons of PVG neurons. Thus, while TNF is expressed in the target tissues of both SCG and PVG neurons, only PVG neurons are capable of responding to target‐derived TNF. These intriguing findings raise several interesting questions for future research. For example, how is TNFR1 expression spatially restricted on SCG neurons and is the expression of TNFR1 on the soma of SCG neurons physiologically relevant?

Although previous work has shown that the reduction in NGF‐promoted axon growth promoted by TNF is mediated by TNFR1 (Kisiswa *et al.*, 2013), TNF reverse signaling can be activated by either TNFR1 or TNFR2 in the immune system (Sun and Fink, 2007). Two observations, however, suggest that TNFR2 does not contribute to the difference between SCG and PVG neurons reported in our study. First, TNFR2 is similarly and weakly expressed in SCG and PVG neurons compared with TNF and TNFR1. Second, TNFR2‐Fc does not affect neurite outgrowth from either SCG or PVG neurons.

The modulation of axon growth by TNF superfamily members, generally without affecting neuronal survival, is becoming an emerging theme in the developing mammalian nervous system. In addition to the opposite effects of TNF forward and reverse signaling on sympathetic axon growth and target innervation discussed here, TNF forward and reverse signaling also exert opposite effects on the growth of dorsal root ganglion axons (Wheeler *et al.*, 2014). In certain populations of neurons, GITRL and APRIL enhance axon growth (O'Keeffe *et al.*, 2008; Osorio *et al.*, 2014; McWilliams *et al.*, 2017), whereas RANKL and LIGHT suppress the axon growth‐promoting actions of neurotrophins (Gavalda *et al.*, 2009; Gutierrez *et al.*, 2013). Reverse signaling mediated by CD40L (McWilliams *et al.*, 2015) and TWEPRIL (Howard *et al.*, 2018) have also been shown to be key physiological regulators of axon growth and tissue innervation in the PNS. Moreover, initial studies in the developing CNS have shown that CD40L‐mediated reverse signaling is a major physiological regulator of dendrite growth and elaboration, having opposite effects on excitatory and inhibitory neurons (Carriba and Davies, 2017).

In summary, our work has revealed unsuspected complexities in the ways in which TNF and TNFR1 contribute to the establishment of sympathetic innervation of different tissues. Axon growth and ramification within different tissues is differentially modulated by TNFR1‐mediated TNF activated forward signaling and by TNF‐mediated TNFR1‐activated reverse signaling in the innervating neurons. These differences are related to differences in the capacity of anatomically different populations of innervating sympathetic neurons to respond to TNFR1 and TNF, which in turn are due, in part, to differences in the cellular distribution of TNF and TNFR1 on responsive neurons.

## Conflict of Interest

The authors declare no competing financial interests.

## Author Contributions

C.E. carried out the background work and performed the whole mount experiments. C.E. and O.Y.C performed the iDISCO and cell culture experiments. O.Y.C. undertook the immuncytochemistry analysis. S.W. carried out the qPCR. L.K. helped with initial preliminary data. A.M.D. and S.W. supervised the project, and A.M.D. wrote the manuscript with input from all authors.

## Availability of Data

The data that support the findings of this study are available from the corresponding author upon reasonable request.
